# Vesicular Glutamate Transporter 3 in age-dependent optic neuropathy

**Published:** 2011-02-05

**Authors:** Gustavo C. Munguba, Andrew S. Camp, Miguel Risco, Mary L. Tapia, Sanjoy K. Bhattacharya, Richard K. Lee

**Affiliations:** Bascom Palmer Eye Institute, University of Miami School of Medicine, Miami, FL

## Abstract

**Purpose:**

To determine retinal vesicular glutamate transporter 3 (VGLUT3) expression alterations in a mouse model of progressive optic neuropathy (glaucoma).

**Methods:**

Tissue specimens were obtained from age-matched DBA/2J and control C57BL/6J mice for western blot analysis. Enucleated globes from DBA/2J, C57BL/6J, and BALB/cJ mice were fixed in formalin, paraffin-embedded, and sectioned for VGLUT3 protein localization.

**Results:**

western blot analysis of the control retinas revealed the expression of a ~55 kDa immunoreactive VGLUT3 protein that is to be expected in tissues such as retina, brain, liver, heart, and kidney tissue, but not in intestinal or lung tissue. Furthermore, a strong ~130 kDa immunoreactive VGLUT3 isoform that is restricted to the central nervous system (the brain and retinas) was also identified in the controls, but was not detected in the DBA/2J retinas. Immunofluorescence microscopy showed a lack of VGLUT3 expression in the synapses between amacrine and retinal ganglion cells in DBA/2J retinas, in contrast to its strong expression in the C57BL/6J and BALB/cJ controls.

**Conclusions:**

Our results implicate the dysregulated expression of a central nervous system-specific VGLUT3 isoform as a predisposing factor in the development of optic neuropathy in DBA/2J mice, a spontaneous mouse model of glaucoma. In striking parallel to the visual system defects of glaucomatous DBA/2J mice, the inner ear of VGLUT3 knockout mice displays a progressive loss of inner hair cell to spiral-ganglion neuron synapses. A significant reduction in the number of spiral-ganglion neurons leads to age-associated deafness. Thus, we propose that the absence of this biochemically uncharacterized 130 kDa VGLUT3 isoform in the DBA/2J retina is a predisposing factor in synaptic instability, and a contributing factor in the age-dependent and progressive loss of ganglion cells projecting to the brain.

## Introduction

Glutamate, the major neurotransmitter in the retina, is loaded into synaptic vesicles by a family of proteins known as vesicular glutamate transporters (VGLUTs) [1]. This protein family consists of three distinct yet highly homologous genes that have defined spatial expression patterns [2]. In the retina, glutamate transporters provide the only means of removing glutamate from the extracellular space and into cells [3] where VGLUTs package this neurotransmitter into vesicles [4]. Although the role of VGLUTs in central nervous system (CNS) exocytic release has been extensively studied [2], VGLUT expression in non-neuronal tissues such as muscle and liver tissue, have led to speculation about additional functions for these vesicular transporters, such as glutamate buffering [5]. It has been suggested that excessive stimulation of the glutamatergic system, “excitotoxicity,” contributes to retinal ganglion cell (RGC) death in glaucoma [3], the second leading cause of irreversible blindness worldwide [6].

In the rodent retina, VGLUT3 is expressed by 1% of the amacrine cell population in the inner plexiform layer (IPL) [7]. Whereas both VGLUT1 and VGLUT2 are expressed by postnatal day 6 (P6) in multiple retinal cell lineages, VGLUT3 is only detected at P10 and is restricted to amacrine, whose dendritic arborization in the IPL expands P15 [8]. The presence of nonglutamate neurotransmitters in VGLUT3-expressing cells, whether amacrine cells [7,9], cholinergic interneurons of the striatum, or serotonin neurons in the raphe [2], is suggestive of nonclassical roles for these neurons [5].

The role of VGLUT3 in these neuronal synapses is still largely unknown, and only recently has VGLUT3 been shown to mediate the regulated exocytosis of glutamate [10]. Furthermore, unlike the limited axonal targeting properties of VGLUT1, VGLUT3 can also target the cell body and dendrites, and has been implicated in the retrograde synaptic release of glutamate [11]. While VGLUT3 expression in the rodent retina is restricted to glycinergic amacrine synapses in the IPL [12], human retinas express VGLUT3 in ganglion cell bodies [9]. This marked contrast in subcellular localization between neighboring neurons of human and rodent retinas raises the question of whether cells expressing nonglutamate neurotransmitters may functionally impart a cell-specific role to VGLUT3.

Deletion of the VGLUT3 gene does not induce compensatory upregulation of other VGLUTs in neurons that are traditionally considered to be nonglutamatergic, such as GABAergic, cholinergic, and serotoninergic neurons. Defects in the VGLUT3 gene have been identified to cause a progressive, age-associated form of high-frequency, non-syndromic deafness (DFNA25), in humans and mice [13]. VGLUT3 knockout (KO) mice, whose inner hair cells (IHCs) lack VGLUT3 expression, and display a progressive loss of IHC to spiral ganglion neuron (SGN) synapses and a significant reduction in the number of SGNs [13]. Furthermore, the ablation of glutamate release through the deletion of the VGLUT3 gene at the superior olive, where the corelease of glutamate by VGLUT3-expressing inhibitory neurons is well established. This results in disrupting developmental synapse elimination and impairing the strengthening of the connections maintained between neurons [14]. In the zebrafish “asteroid” mutant, the absence of VGLUT3 results in the reduction of synaptic vesicles at ribbon bodies in IHCs and abolishes post-synaptic action currents [15]. The importance of VGLUT3 expression in the auditory system, which has many structural and functional analogs to the visual system, raises the possibility that this vesicular glutamate transporter plays a nonredundant role in the retina.

The functional role of VGLUT3 in the retina remains largely unknown. We addressed this issue by studying the DBA/2J mouse glaucoma model of age-associated, chronic progressive optic neuropathy and RGC loss [16]. These mice develop age-dependent progressive eye abnormalities, including elevated intraocular pressure starting at 8–9 months, severe optic nerve damage, and significant RGC loss by 12 months of age [16-20]. Probing of retinal neuron population changes in these mice originally revealed an exclusive loss of RGCs [21]; however, recent studies report changes in the GABAergic and cholinergic amacrine cell populations, whereas glycinergic amacrine cells, which are known to express VGLUT3 [12], are unaffected [22]. Large studies categorizing DBA/2J animal pathology have shown optic nerve degeneration that is variable, asymmetric, and progressive, leading to optic nerve head excavation secondary to RGC death, a characteristic feature of glaucoma [17].

Here, we report on impaired retinal VGLUT3 expression in an inbred mouse optic neuropathy model associated with RGC loss. VGLUT3 expression is absent in synapses between amacrine cells and RGCs in DBA/2J mice. Our findings implicate the dysregulated expression of a CNS-specific VGLUT3 isoform as a predisposing factor in the development of optic neuropathy in DBA/2J mice. In striking parallel to the visual system defects of glaucomatous DBA/2J mice, the inner ear of VGLUT3-KO mice displays a progressive loss of IHC to SGN synapses, and a significant reduction in the number of SGNs, leading to age-associated deafness [13]. Thus, we hypothesize that the absence of this biochemically uncharacterized 130 kDa VGLUT3 isoform in the DBA/2J retina is a predisposing factor in synaptic instability, and an age-dependent, progressive contributing factor in the loss of ganglion cells projecting to the brain. Furthermore, we identify a component of DBA/2J retina-specific pathology and a biochemically isoform type of VGLUT3 that is CNS-restricted, both previously uncharacterized, to our knowledge.

## Methods

### Animals

DBA/2J, C57BL/6J, and BALB-cJ animals were bred and handled according to the ARVO Statement for the Use of Animals in Ophthalmic and Vision Research. All animal procedures were approved by the University of Miami Institutional Animal Care and Use Committee.

### Immunofluorescence and microscopy

The primary antibodies used were guinea pig anti-VGLUT3 (AB5421, 1:4,000 dilution; Chemicon, Temecula, CA) and guinea pig anti-VGLUT1 (AB5905, 1:1,000 dilution; control not shown in figures; Chemicon). The secondary antibody used was donkey antiguinea pig IgG Cy5 (706–175–148, 1:100 dilution; Jackson Labs, West Grove, PA). Globes or brains were fixed using 4% formalin (P1648; Sigma, St. Louis, MO) in 1X phosphate buffered saline (PBS; 127 mM NaCl, 2.7 mM KCl, 10 mM phosphate) buffer at 25 °C. Following enucleation or dissection and fixation, globes and brains were embedded in paraffin, sectioned, and mounted on glass slides. We had sectioned and immunostained slides covering the entire length of the retina. Each section was cut to a 10 um thickness, and one out of every 10 slides was used to walk along the entire retina of two 8-month-old C57BL/6J and DBA/2J animals. We also immunostained retinas from at least three different animals at each given age for C57BL/6J and DBA/2J mouse strains, always comparing representative areas of both the DBA/2J and C57BL/6J control animals. The age groups used for the C57BL/6J and DBA/2J animals were 9 days, 3 months, 8 months, 12 months, and 19 months. Two BALB/cJ animals were used as an additional positive control at three months of age. Brains from two 8-month-old C57BL/6J and DBA/2J animals were also used as additional controls for immunostaining. Slides were rehydrated in xylene and sequential alcohol rinses, then treated at 95 °C with Trilogy (CMX833-C; Cell Marque, Rocklin, CA) antigen retrieval reagent. Tissue sections were subsequently blocked with Rodent Block M (RBM96; BioCare Medical, Concord, CA) for 30 min to reduce nonspecific binding. Slides were incubated overnight at 4 °C in PBS containing primary antibodies, washed with PBS, incubated in antibody buffer containing secondary antibodies for 1 h at room temperature, washed with PBS, and mounted onto glass coverslips using Vectashield with DAPI (Vector, Burlingame, CA). Images were collected on an inverted microscope (Axiovert 200M; Carl Zeiss Meditec, Oberkochen, Germany) running the Zeiss AxioVision 4.7.2 on PC (Zeiss Inc., Thornwood, NY).

### Western blot analysis

Tissues were homogenized in radio-immunoprecipitation assay (RIPA) buffer (50 mM Tris-HCl, pH 8.0, 150 mM NaCl, 1% Igepal, and 0.5% sodium dodecyl sulfate, and 0.5% deoxycholic acid) with complete miniprotease inhibitors (11836153001; Roche, Indianapolis, IN) using a mechanical homogenizer (Polytron PT1200; kinematica Inc. Cincinnati, OH). Retinas were homogenized in RIPA buffer with complete miniprotease inhibitors using a disposable Kontes Pellet Pestle with cordless motor tissue grinder (Kimble Kontes, Vineland, NJ). Following homogenization, tissues were passed through a 21-gauge needle six times, and the soluble fraction was used as the protein lysate. The insoluble fractions were also checked by western blot analysis (not shown in figures). Twenty micrograms of protein were loaded per well for each sample. The primary antibody used was rabbit anti-VGLUT3 (AB23977, 1:2,500 dilution; Abcam, Cambridge, MA). The secondary antibody used was peroxidase-conjugated AffiniPure Donkey antiguinea pig IgG (H^+^L; 706–035–148, 1:10,000 dilution; Jackson Labs). The membranes were blocked for 1 h with phosphate-buffered saline with 0.1% Tween-20 (PBST) containing 5% milk, incubated overnight at 4 °C in 2% milk in PBST containing primary antibodies, washed with PBST, incubated in 2% milk in PBST containing secondary antibodies for 30 min at room temperature, and washed with PBST and PBS. The chemiluminescent substrate used was SuperSignal West Pico (34080; Thermo Scientific, Rockford, IL).

## Results

### A 130 kDa central nervous system specific isoform of VGLUT3 is undetectable in DBA/2J retinal tissue

**Figure 1:**
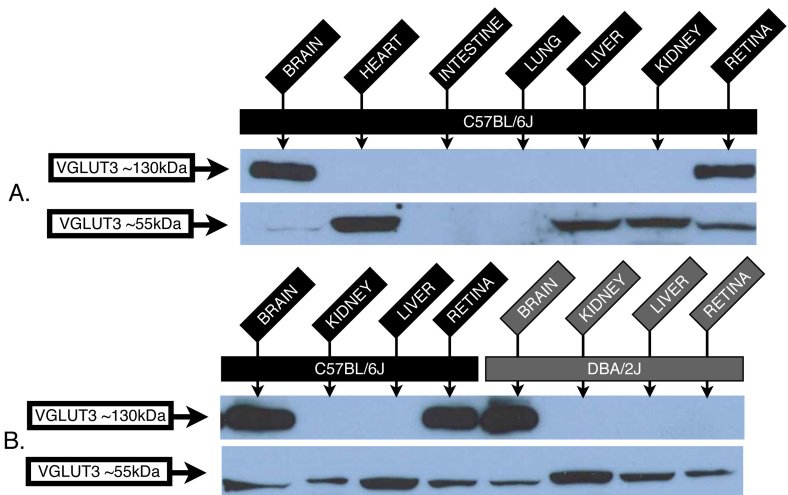
Central nervous system-specific 130 kDa isoform of vesicular glutamate transporter 3 that is undetectable in DBA/2J retina. Western blot analysis of C57BL/6J mouse lysates demonstrate expression of an expected vesicular glutamate transporter 3 (VGLUT3) 55 kDa immunoreactive protein band in brain, heart, liver, kidney and retina tissue, and a strong 130 kDa band restricted to the central nervous system (brain and retina tissue; **A**). Immunoreactivity of the 130 kDa VGLUT3 band is undetectable in DBA/2J retinas, while present in DBA/2J brains (**B**). The 55 kDa VGLUT3 isoform serves as an internal loading control. Expression levels of the 55 kDa VGLUT3 protein in all tested tissue samples (including the retina) were comparable to control C57BL/6J animal tissues (**B**). All wells were loaded with 20 μg of protein from age-matched eight-month-old animals.

VGLUT3 western blot analyses of the control C57BL/6J mice demonstrated the expression of an expected ~55 kDa immunoreactive protein in tissues such as retina, brain, and liver tissue, while expression was absent from intestine and lung tissue (Figure 1A). This VGLUT3 expression pattern was consistent with previous mRNA (mRNA) expression studies. We further showed strong VGLUT3 protein expression in the heart and moderate expression in the kidney.

Surprisingly, a strong ~130 kDa immunoreactive VGLUT3 isoform was seen in both the brain and retina tissue (Figure 1A). This 130 kDa band was substantially brighter than the expected 55 kDa VGLUT3 band, and was restricted to CNS tissues. Furthermore, the comparison of DBA/2J and control C57BL/6J lysates showed differences in VGLUT3 expression in the retina. DBA/2J retinas lacked expression of the CNS-specific isoform of VGLUT3, based upon the western blot analysis, while robust expression of this isoform in DBA/2J brain was observed (Figure 1B). While DBA/2J mice selectively expressed the CNS-restricted 130 kDa isoform only in the brain, the expected 55 kDa VGLUT3 band showed no change in expression in all the other tissues assayed, including retina tissue (Figure 1B).

### VGLUT3 amacrine cell synapses are undetectable in DBA/2J retinas

The expression pattern of VGLUT3 in the retina was assessed by immunofluorescence. A lack of any VGLUT3 immunofluorescence signal specific to the IPL of the DBA/2J retinas was corroborated by the absence of expression found by western blot analysis of the 130 kDa VGLUT3 isoform.

**Figure 2:**
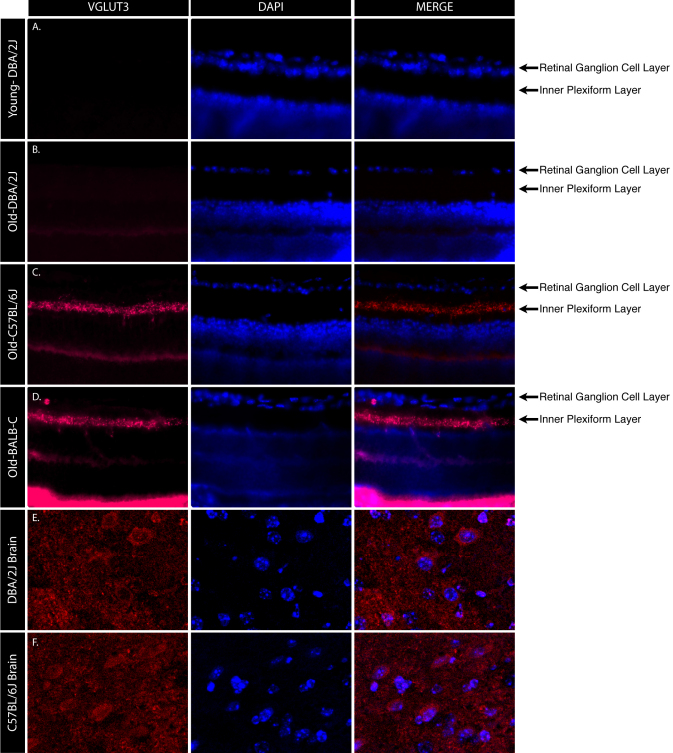
Vesicular glutamate transporter 3 amacrine cell synapses that are undetectable in DBA/2J retinas. Representative globe cross-sections immunostained with vesicular glutamate transporter 3 (VGLUT3) antibody show VGLUT3 expression in the retinal inner plexiform layer. Immunostaining shows VGLUT3 (red) expression in the control C57BL/6J (**C**), and BALB/c (**D**) retinas, while young nonglaucomatous DBA/2J (**A**) or old glaucomatous DBA/2J (**B**) VGLUT3 expression levels are undetectable. Nuclear DAPI staining (blue) is also shown. Merge represents the combination of Blue and Red channels. Expression was assessed in animals ranging from postnatal day 8 to 21 months of age. Immunostaining of DBA/2J (**E**) and C57BL/6J (**F**) tissue shows VGLUT3 (red) expression in the brain of both animal strains.

We assessed whether or not the lack of DBA/2J VGLUT3 immunostaining had a different temporal pattern by immunostaining DBA/2J retinas from as early as P9 to as late as 19 months of age. Sections along the entire length of the retina were analyzed for both DBA/2J and C57BL/6J globes. VGLUT3 immunostaining was undetectable at any cross-section along the DBA/2J retinas at all ages examined, whereas robust VGLUT3 immunostaining was observed in all cross-sections along the IPL of the control C57BL/6J and BALB/cJ retinas (Figure 2A-D). Immunostaining of DBA/2J and C57BL/6J brain slices showed robust VGLUT3 immunoreactivity in both strains (Figure 2E,F).

## Discussion

Our data show that VGLUT3 expression can be modulated in a tissue-specific manner providing the CNS (retina and brain) with a unique, and up to now uncharacterized, 130 kDa isoform of VGLUT3, in addition to the known 55 kDa isoform of VGLUT3. The absence of the CNS-specific VGLUT3 isoform in DBA/2J retinas, but not in the brain, also demonstrates that VGLUT3 isoforms in brains and retinas are modulated independently. Our results suggest that this previously uncharacterized high molecular weight, CNS-specific, VGLUT3 isoform is the primary VGLUT3 species residing at CNS synapses. This result is corroborated by the lack of a VGLUT3 immunofluorescence signal specific to the inner plexiform layer of DBA/2J retinas, in association with the lack of expression of the 130 kDa VGLUT3 isoform found by western blot analysis.

Work by Schafer and colleagues, who cloned and characterized VGLUT3 from P1 mouse brain tissue, attribute several broad protein bands observed by western blot analysis to the presence of possible dimerization products or nonspecific aggregation or processing artifacts from vaccinia-infected cells. In their work, Schafer et al. [18] identify glycosylation and phosphorylation sites, suggesting the possibility that posttranslational modifications contribute to this higher molecular weight isoform of VGLUT3. Alternative splicing of the VGLUT3 gene is yet another explanation that cannot be excluded. In contrast to our finding of VGLUT3 protein expression in the kidney, northern blot analysis experiments by Schafer and colleagues revealed no VGLUT3 hybridization in a 4.9 kb mRNA probe of kidney mRNA, while observing several liver-derived mRNA species, thereby supporting the likelihood of alternative splicing as an explanation for the 130 kDa VGLUT3 isoform [23].

Focal VGLUT3 expression in the retina has previously been attributed to synaptic vesicles in subpopulations of amacrine cells [5,7,12]. Johnson and colleagues observed the presence of VGLUT3 retinal protein beginning at P8, which achieved an adult expression pattern by P12 [12]. The 55 kDa isoform of VGLUT3, but not the 130 kDa isoform, is detectable in DBA/2J retinas by western blot analysis. Although DBA/2J retinas express the lower molecular weight VGLUT3 isoform, immunofluorescence signals are undetectable at amacrine cell synapses. Furthermore, immunostaining of DBA/2J brain slices show the robust immunoreactivity of VGLUT3, corroborating the presence of the 130 kDa isoform by western blot analysis. These data suggest VGLUT3 species may play distinctive roles in different cell types. In association with observed changes in amacrine cell populations of DBA/2J animals, it is possible that the VGLUT3 amacrine cell population may be entirely absent in DBA/2J retinas, compared to other mouse strains. This is unlikely, however, because comparisons between DBA/2J and C57BL/6J mouse retinas either have suggested cell loss restricted to the RGC layer [21] or have excluded any changes to glycinergic amacrine cells, previously identified as a VGLUT3 amacrine cell [22] subpopulation in the DBA/2J strain [12].

The inner ear has striking similarities to the retina. IHCs of the inner ear function as mechanoelectrical transducers conveying auditory signals through SGNs (analogous to RGCs in the retina) to the CNS. SGNs receive auditory signals from IHCs and relay these signals to the brain via long axons consisting of the eighth cranial nerve. While spiral ganglia express N-methyl-D-aspartate (NMDA), α-amino-3-hydroxyl-5-methyl-4-isoxazole-propionate (AMPA), and kainate receptors, the functional distinction of individual receptors and their roles, using selective AMPA receptor agonists and other methods, suggests a predominant role for AMPA receptors in IHC-mediated neurotransmission to the CNS, thereby implicating glutamate as an important neurotransmitter in auditory neurotransmission [24]. The expression of various receptors by spiral ganglia is not surprising, considering that VGLUT3 is the only VGLUT expressed by IHCs that signals directly to SGNs. Like neurons in the retina, auditory neurons are also sensitive to glutamate-mediated excitotoxicity, and are also dependent on active synaptic inputs for survival.

Defects in the VGLUT3 gene have been identified the cause of a progressive, age-associated form of high-frequency nonsyndromic deafness, DFNA25 [13]. VGLUT3-KO mice, whose IHCs lack VGLUT3 expression, display a progressive loss of IHC-to-SGN synapses, and a significant reduction in the number of SGNs [13]. Interestingly, age-related, progressive hearing loss has also been implicated in the DBA/2J progressive glaucoma model [25]. DBA/2J animals possess the ahl mutation cadherin 23 [25] and a fascin-2 gene mutation [26] whose combined effect results in early-onset hearing loss [27,28]. Because age-related hearing loss is a multifactorial disease process, with many similarities to glaucomatous optic neuropathy, it is tempting to speculate that the lack of the CNS-specific 130 kDa VGLUT3 isoform in the visual system might also repeat itself in the auditory synapses, providing yet another contributor to progressive hearing loss, though this has not yet been investigated. Parallels between the progressive loss of glaucomatous DBA/2J RGCs and the progressive loss of the auditory CNS-projecting SGNs of VGLUT3-KO mice may be indicative of similar molecular pathophysiological mechanisms. In both cases, perturbation of VGLUT3 integrity in cells making direct synapses to CNS-projecting ganglia—RGCs in glaucoma and SGNs in DFNA25 progressive deafness—is associated with a progressive loss of synaptic stability and results in age-related and irreversible first-order neuron loss.
